# Molecular diagnosis of suspected intestinal schistosomiasis in a non-endemic area of Yunnan Province, China

**DOI:** 10.1186/s40249-025-01372-y

**Published:** 2025-10-09

**Authors:** Shiyuan Wu, Chunhong Du, Chunli Cao, Jihuang Yang, Fang Luo, Xiaolin Ma, Qing Hu, Minwei Yuan, Yun Zhang, Zongya Zhang, Zaogai Yang, Changchun Gou, Li Wang, Jizhou Han, Shizhu Li, Yi Dong, Jipeng Wang

**Affiliations:** 1 State Key Laboratory of Genetics and Development of Complex Phenotypes, Ministry of Education Key, Laboratory of Contemporary Anthropology, School of Life Sciences, Fudan University, Shanghai, 200438 China; 2 Yunnan Key Laboratory for Zoonosis Control and Prevention, Yunnan Institute for Endemic Diseases Control and Prevention, Kunming, 650500 China; 3 National Center for International Research On Tropical Diseases, National Institute of Parasitic Diseases at Chinese Center for Disease Control and Prevention, Shanghai, 200025 China; 4 Dehong Prefecture Center for Disease Control and Prevention, Mangshi, 678400 Yunnan China; 5 Dehong Prefecture People’s Hospital, Mangshi, 678400 Yunnan China; 6 College of Life Sciences, Inner Mongolia University, Hohhot, Inner Mongolia Autonomous Region China; 7 State Key Laboratory of Estuarine and Coastal Research, East China Normal University, Shanghai, 200241 China; 8 Division of Life Sciences and Medicine, University of Science and Technology of China, Hefei, 230027 Anhui China; 9 Molecular Archaeology Laboratory, Fudan University, Shanghai, 200433 China; 10 Kunming Medical University, Kunming, Yunnan 650500 China; 11 Mangshi Center for Disease Control and Prevention, Dehong Prefecture, Yunnan 678400 China

**Keywords:** Formalin and embedded in paraffin blocks, Micro-library construction, Next-generation sequencing, Schistosomiasis, Yunnan

## Abstract

**Background:**

While *Schistosoma japonicum* is endemic in the Yangtze River Basin and parts of Yunnan and Sichuan provinces in China, Mangshi City in Dehong Prefecture, Yunnan Province, is not recognized as an endemic area. Between 1996 and 2018, more than 20 suspected schistosomiasis cases were reported in this region. Despite clinical symptoms consistent with *S. japonicum* infection, no eggs were detected in feces, and the intermediate host *Oncomelania hupensis* was absent locally. Most patients had no travel history to known endemic areas, leaving the infections unconfirmed.

**Findings:**

Rectal biopsy specimens from four suspected cases, preserved as formalin-fixed paraffin-embedded (FFPE) tissues, were re-examined. Microscopy revealed egg-like structures resembling *Schistosoma* spp. in the specimens. Due to severe DNA degradation from long-term storage (6–16 years), a micro-library construction method was applied to two samples to enable next-generation sequencing (NGS). Using two independent alignment strategies, multiple sequence reads corresponding to *S. japonicum* were identified in both samples, providing molecular confirmation of infection.

**Conclusions:**

This study presents the first molecular evidence confirming *S. japonicum* infection in a non-endemic area of Yunnan Province. The findings highlight the diagnostic potential of combining FFPE samples with NGS to resolve long-standing suspected cases lacking conventional parasitological evidence and underscore the importance of continued surveillance in regions not currently classified as endemic.

**Graphical Abstract:**

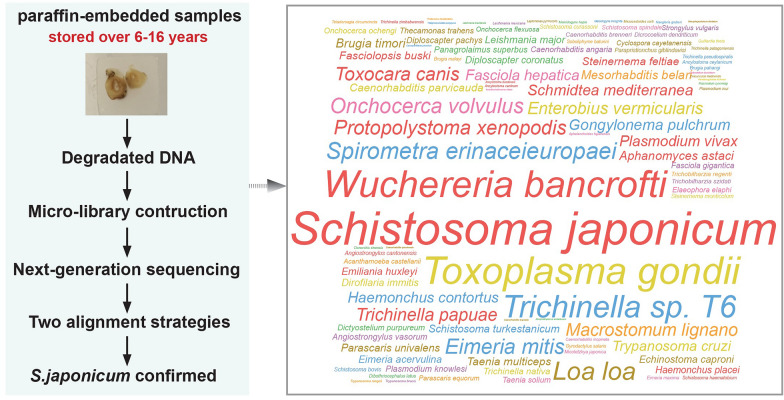

**Supplementary Information:**

The online version contains supplementary material available at 10.1186/s40249-025-01372-y.

## Background

Schistosomiasis japonica, caused by *Schistosoma japonicum*, remains endemic in 18 counties of Yunnan Province, China, including regions in Dali, Lijiang, Chuxiong, and Honghe. However, Mangshi City in Dehong Prefecture is not officially recognized as endemic. Between 1996 and 2018, over 20 clinically suspected cases were reported in Mangshi, primarily diagnosed based on rectal biopsy and ultrasonography (Additional file 1). Most patients were local residents without a travel history to endemic regions, and stool examinations failed to detect *S. japonicum* eggs, leaving the infections unconfirmed. Zhefang Town, located in southwestern Mangshi and bordering Myanmar, is an agriculturally dominant region with a population of over 50,000. It lies within a flat intermountain basin, traversed by the Mangshi River and its tributaries. The area has a tropical monsoon climate and favorable ecological conditions for the intermediate snail host *Oncomelania hupensis*. Despite this, no snail habitats have been identified locally.

The first suspected case in Mangshi (formerly Luxi City) was reported in 1998 [1]. Liver and rectal biopsies revealed calcified *S. japonicum* eggs, but no eggs were found in stool. This prompted further investigations by local Center for Disease Control and prevention (CDC) and academic partners, identifying three additional cases confirmed by histopathology [2]. All patients denied contact with known transmission sites, raising the possibility of local or cross-border transmission. From 2008 to 2013, serological screening of 1331 residents in Zhefang Town revealed that 66 individuals (4.96%) were positive for schistosome antibodies [3]. Sixteen had liver damage on ultrasound, and 10 had schistosome eggs confirmed by rectal biopsy. Notably, none had traveled to endemic areas, although some had lived in Myanmar, where schistosomiasis is re-emerging. Most cases were adult males, suggesting a possible occupational or environmental exposure risk. Despite these findings, no cases of acute schistosomiasis or portal hypertension were reported, and the absence of *O. hupensis* undermines evidence for active local transmission. Thus, two main limitations have hindered confirmation of transmission: the absence of the snail host and a lack of molecular confirmation of the pathogen.

Given that most cases are historical and follow-up is not feasible, alternative strategies are needed. One such approach involves using archived formalin-fixed paraffin-embedded (FFPE) biopsy tissues, which are commonly used in clinical diagnostics and may still contain pathogen DNA [4]. Although FFPE samples preserve tissue morphology, formalin fixation degrades DNA, and long-term storage further reduces nucleic acid quality [5, 6]. Previous studies have shown that next-generation sequencing (NGS) can be performed on FFPE samples, particularly using micro-library preparation methods to accommodate fragmented DNA [7, 8]. However, successful sequencing of parasite DNA from FFPE samples is rarely reported, and no published studies have confirmed schistosomiasis using this method. Therefore, this study aimed to extract nucleic acids from rectal FFPE biopsy samples of suspected schistosomiasis patients and conducted NGS sequencing to identify potential pathogens at the molecular level.

## Methods

### FFPE samples sources

The samples, which were stored in 1997 (male, born in 1932, deceased), 2008 (male, born in 1951), 2011 (male, birth year unknown), and 2018 (male, born in 1932, deceased), were obtained from archived specimens housed in the Pathology Department of Dehong Prefecture Hospital, Yunnan Province. Given the retrospective nature of this study, the extended storage periods (14 and 17 years) for samples from surviving individuals, and the advanced age of those subjects, follow-up was not feasible. Therefore, the use of these specimens was not associated with any ethical concerns.

### DNA extraction from FFPE blocks

A portion of the tissue was removed from the paraffin-embedded blocks, and genomic DNA was extracted using a modified Cetyltrimethylammonium Bromide (CTAB) method based on a previously published protocol [9]. Briefly, 600 μl of 2% CTAB (Alladin, Shanghai, China) solution was added to a 1.5 ml centrifuge tube, followed by 30 μl β-mercaptoethanol and a small portion of the sample. The mixture was vortexed, and 20 μl of proteinase K was added. The samples were incubated at 65 °C for 1 h. Subsequently, 500 μl of phenol: chloroform: isoamyl alcohol (25∶24∶1) was added, vortexed, and centrifuged at 16,200 × *g* for 15 min at low temperature. The supernatant was collected, and 500 μl of chloroform: isoamyl alcohol (24∶1) was added. The mixture was vortexed, centrifuged at 16,200 × *g* for 15 min, and the supernatant was collected. To the supernatant, 500 μl of isopropanol was added and mixed by inversion. The samples were then frozen at −80 °C for 30 min, centrifuged at 16,200 g for 15 min, and the supernatant was discarded, leaving the DNA pellet. The pellet was washed twice with 75% ethanol, and any residual liquid was removed. The DNA was dissolved in H₂O based on the pellet size.

### Micro-library construction and sequencing

Extracted DNA was used to construct double-stranded DNA libraries following the protocol described in a previous study [10]. Index primers were incorporated, followed by PCR amplification using Q5 High-Fidelity DNA Polymerase (NEB, Massachusetts, USA) for up to 17 cycles. The resulting indexed libraries were purified with AMPure XP Beads (Beckman Coulter, Californi, USA) and subsequently sequenced on the Illumina platform (PE100).

### Sequence alignment methods

Two methods were used for sequence analysis:

Method 1: Assembly of Sequences. First, AdapterRemoval was used to identify and remove sequencing adapter sequences, as well as to trim N bases and low-quality bases at the ends of the reads. For paired-end reads, overlapping regions between paired sequences were merged to generate single combined sequences, retaining only those longer than 30 bp. The fragment length distribution was visualized using R (https://www.r-project.org/). Subsequently, MEGAHIT (https://github.com/voutcn/megahit) was employed to perform de novo assembly on the merged single-end reads, and contigs shorter than 300 bp were filtered out.

Method 2: Direct sequence analysis. Data quality was controlled by filtering out low-complexity and low-quality sequences. Human genomic sequences (hg38 reference genome) were removed. The remaining fragments were then aligned to the genomes of 179 helminth species obtained from WormBase Parasite (https://parasite.wormbase.org/species.html) and 96 protozoan species from NCBI.

Sequences were trimmed using fastp (https://github.com/OpenGene/fastp) to remove poly-X tails, filter low-complexity sequences, and discard low-quality nucleotides. String Graph Assembler (Jared Simpson, Ontario, Canada) was applied for de-duplication, where duplicates were defined as reads either identical to or contained within another read. Host contamination was removed by mapping the sequences to the human genome using burrows-wheeler-alignment tool (http://bio-bwa.sourceforge.net/). The remaining reads were aligned to a comprehensive database of eukaryotic and parasitic genomes using Bowtie2 (Ben Langmead, Maryland, America).

For taxonomic classification, the Next-Generation Sequencing Lowest Common Ancestor (https://github.com/miwipe/ngsLCA) algorithm was applied to DNA reads aligned to multiple organism reference databases. Parameters used included “-simscorelow 0.9, -simscorehigh 1.0, -editdistmin 0, -editdistmax 10, -minmapq 0, -fix_ncbi 0.” The classification leveraged the NCBI taxonomy and resolved multiple alignments using the lowest common ancestor approach. Data visualization was performed using MEGAN (https://www.wsi.uni-tuebingen.de/lehrstuehle/algorithms-in-bioinformatics/software/megan6/).

### Microscopic imaging

Microscopic imaging of rectal biopsy specimens stained with hematoxylin and eosin (HE) was performed using a Nikon ECLIPSE Ts2-FL microscope. Measurements of egg size were obtained using the associated image processing software.

### Statistical analysis

In this study, GraphPad Prism 9 software (GraphPad Software Inc., San Diego, CA, USA) was utilized for all data visualization and statistical analyses. Continuous variables with normal distribution were presented as mean ± standard deviation (SD), while non-normally distributed variables were reported as median (interquartile range). For categorical data, chi-square tests were employed. A significance level of *P* < 0.05 was set for all analyses.

## Results

**Fig. 1:**
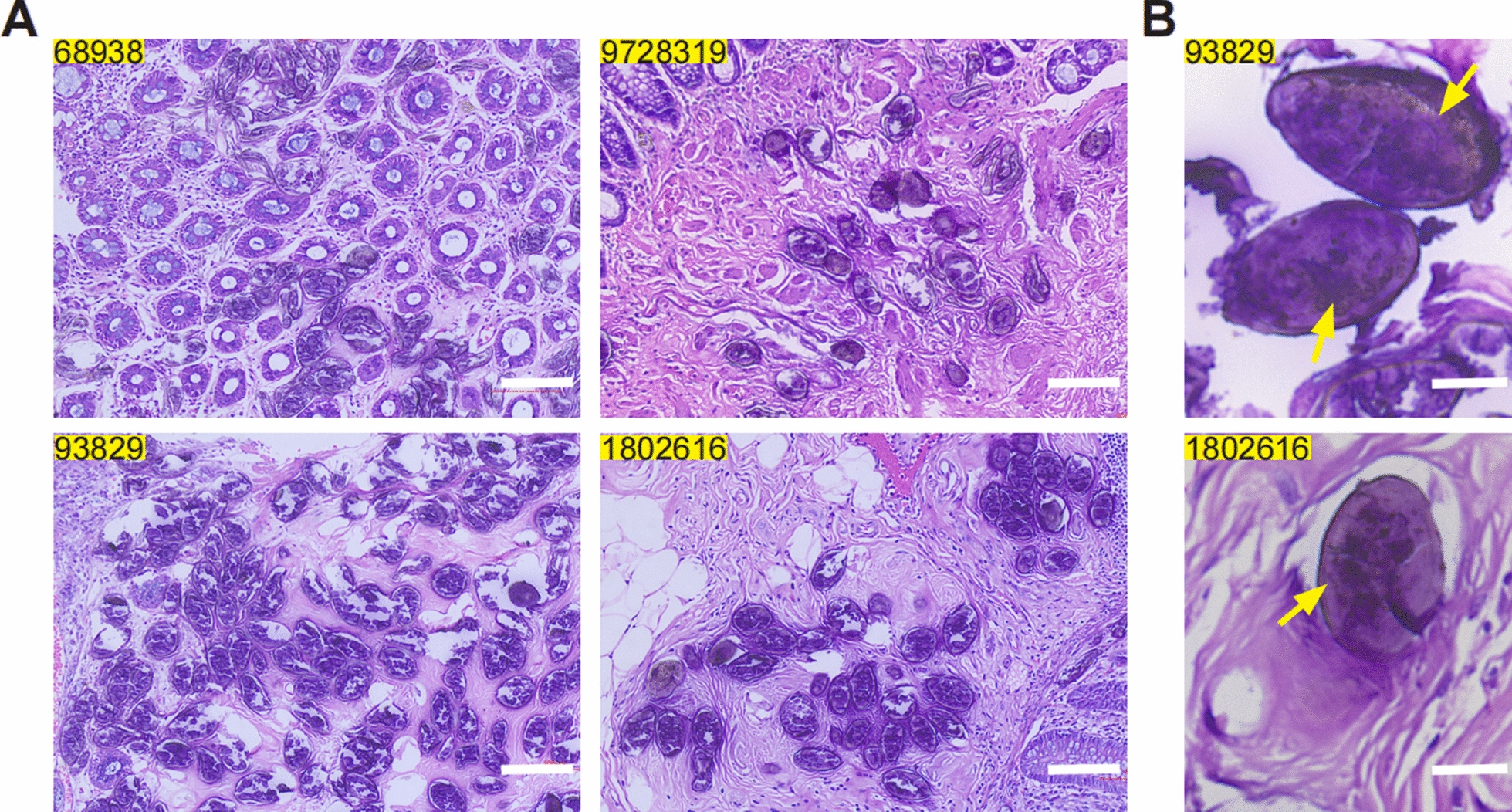
Rectal stained tissue section examination of suspected schistosomiasis cases in Mangshi, Dehong Prefecture, Yunnan Province. **A** Morphology of the eggs in rectal stained tissue sections from four suspected cases. Scale bars: 100 µm. **B** Morphology of intact eggs (indicated by yellow arrow) in some sections. Scale bars: 25 µm

Rectal biopsy specimens were obtained from four patients with suspected *S. japonicum* infection. HE-stained sections revealed structures morphologically consistent with *Schistosoma eggs* in all cases, without evidence of periegg calcification (Fig. 1A). Egg measurements ranged from 60.803 to 63.927 µm in length and 36.918–42.476 µm in width (Table 1), slightly smaller than the dimensions typically reported for *S. japonicum* eggs [11]. In several samples, intact oval-shaped eggs were observed, with small terminal spines visible in some instances (Fig. 1B).

**Table 1:** Egg size statistics of suspected schistosomiasis cases in rectal tissue sections from Yunnan, China

Sample ID	Egg length (µm)	Egg width (µm)
68938	60.803±9.801	37.979±8.191
93829	62.987±7.400	36.918±5.602
1802616	63.927±8.341	39.112±5.993
9728319	62.479±9.137	42.476±8.003

Epidemiological assessment indicated that all patients resided in areas not endemic for *S. japonicum* and lacked classic pathological features of schistosomiasis. Consequently, the cases remained unconfirmed and were classified as “suspected schistosomiasis”. The pathology specimens had been stored for 6–16 years, with preparation dates as follows: 68938 (2008), 9728319 (1997), 93829 (2011), and 1802616 (2018).

**Fig. 2:**
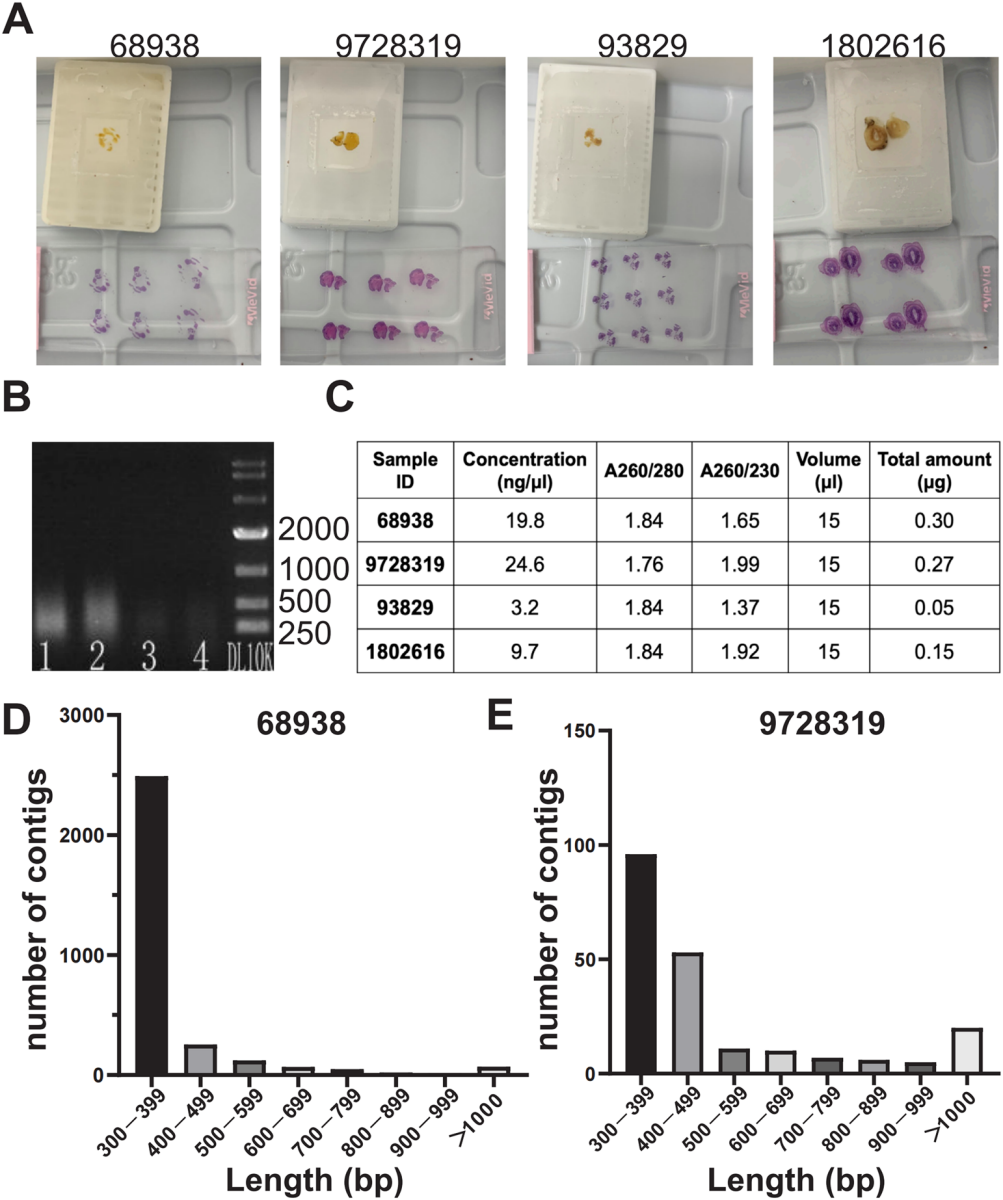
DNA extraction and library construction sequencing of paraffin-embedded rectal samples from suspected schistosomiasis cases. **A** Paraffin blocks and stained tissue sections of the four suspected schistosomiasis cases. **B** Gel electrophoresis results of DNA extracted from the four samples. 1, 68938; 2, 9728319; 3, 93829; and 4, 1802616. **C** DNA concentration and quality of the four samples. **D** Length distribution of long fragments (> 300 bp) after sequencing and assembly of sample 68938. **E** Length distribution of long fragments (> 300 bp) after sequencing and assembly of sample 9728319

DNA was extracted from FFPE tissue blocks of these four suspected cases (Fig. 2A). Gel electrophoresis demonstrated extensive DNA degradation (< 1,000 bp) and low yields (Fig. 2B, C). Only samples 68938 and 9728319 exceeded the 300 ng threshold for library preparation (Fig. 2B, C). Conventional library construction failed for sample 9728319 because of severe fragmentation. To address this, we applied a micro-library construction strategy, which successfully generated sequencing libraries from both high-yield samples. Sequencing yielded short DNA fragments typical of degraded FFPE samples, with mean read lengths of 59.2 bp (sample 68938) and 40.4 bp (sample 9728319) (Supplementary Fig. 1).

De novo assembly produced longer contigs: 2977 sequences > 300 bp for sample 68,938 and 220 for sample 9728319 (Additional file 2–5). As shown in Fig. 2D, E, most of the assembled sequences ranged between 300 and 500 bp. nBLAST analysis of sample 68938 identified 2578 sequences (86.6%) as human-derived and 156 with highest similarity to *S. japonicum*, including 13 perfectly matching reference sequences in public databases (Additional file 4). These included the *S. japonicum 28S rRNA gene* (Z46504.4), *S. japonicum isolate S5 small subunit ribosomal RNA gene* (OQ884000.1), *S. japonicum isolate SCXC06 mitochondrion* (KU196392.1), *S. japonicum isolate YNEY07 mitochondrion* (KU196414.1), *S. japonicum isolate Anhui clone BAC C108_24K02* (FN293023.1), and *S. japonicum isolate Anhui non-coding mRNA clone SJFCE1965|FSE001-P00033-F06* (FN328762.1). Sample 9728319 contained 114 human-derived sequences (51.8%) and 17 *S. japonicum*-like sequences, four of which were identical to known *S. japonicum* sequences: *S. japonicum 28S rRNA gene* (Z46504.4), *S. japonicum isolate SJJYM3 28S ribosomal RNA gene* (EU835685.1), *S. japonicum isolate Anhui clone BAC C108_72G12* (FN293030.1), *S. japonicum clone ZZD247 mRNA sequence* (AY223209.1) (Additional file 5).

These findings confirm that both samples contained multiple sequences perfectly matching the *S. japonicum* genome, thereby establishing a molecular diagnosis of *S. japonicum* infection.

**Fig. 3:**
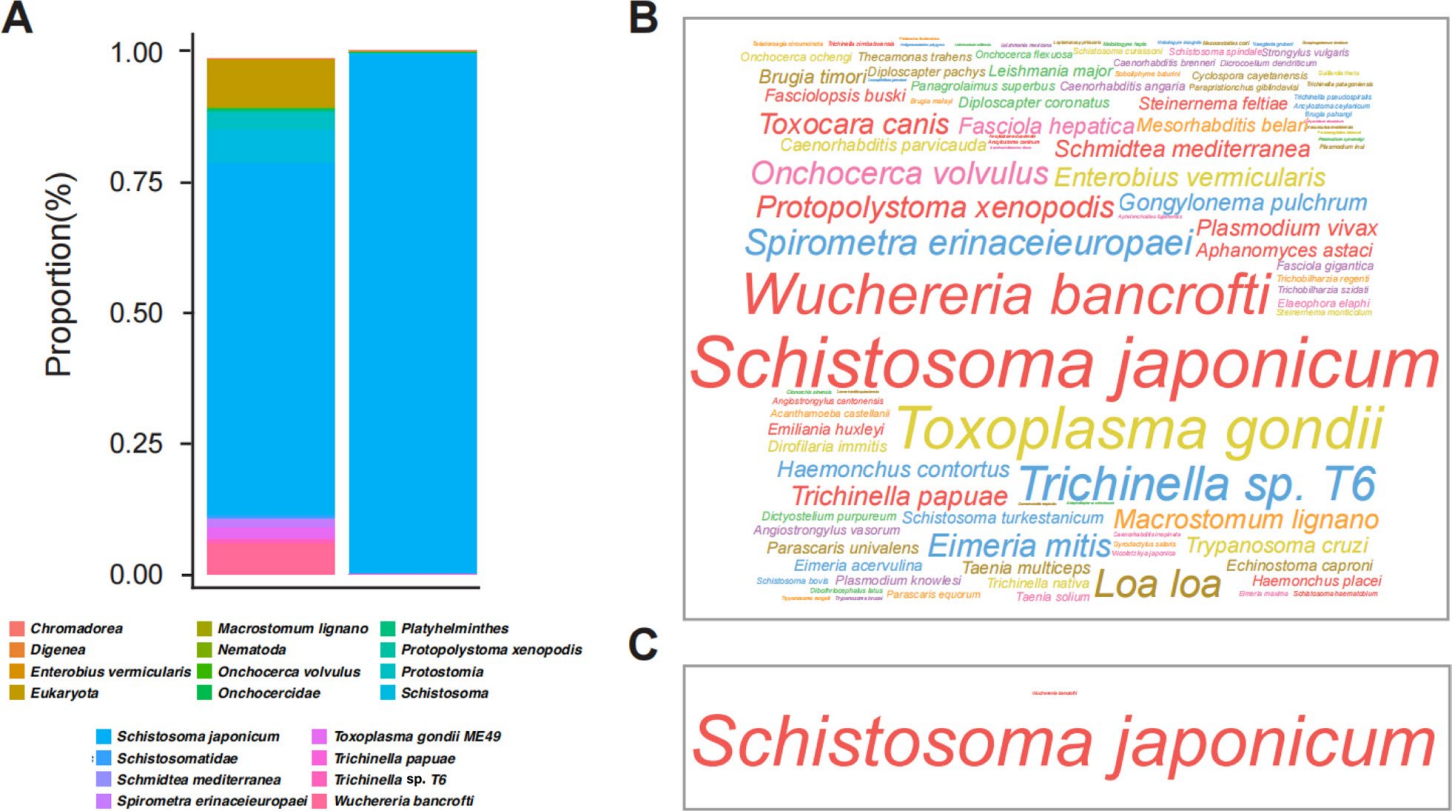
Sequence alignment of sequencing data from paraffin-embedded rectal samples of suspected schistosomiasis cases. **A** Proportion of sequencing reads from two suspected schistosomiasis cases aligned to different species in the eukaryotic database. **B** Weight distribution of sequencing data from sample 68938 aligned to different species. **C** Weight distribution of sequencing data from sample 9728319 aligned to different species. In panels **B** and **C**, the larger the font, the higher the weight represented

Because most assembled sequences originated from host DNA (Additional file 4 and 5), we employed an alternative bioinformatic pipeline to enhance parasite detection. Low-quality and low-complexity reads were filtered, human sequences removed, and the remaining reads aligned directly to eukaryotic and parasitic genomes (Additional file 6). Using this approach, over 80% of the filtered sequencing reads in both samples aligned to the *Schistosoma* genome (Fig. 3A). Taxonomic classification confirmed that *S. japonicum* was the predominant species, (Fig. 3B, C), with no cross-alignment to other *Schistosoma* species.

These two complementary approaches conclusively identified *S. japonicum* DNA in two long-preserved FFPE rectal biopsies, establishing a definitive diagnosis despite the patients residing in a non-endemic region.

## Discussion

Between 1996 and 2018, over 20 suspected cases of schistosomiasis were reported in Mangshi City, Yunnan Province—an area not recognized as endemic for *S. japonicum* in China. These suspicions were based on findings from rectal biopsies and ultrasonography. However, subsequent epidemiological investigations failed to detect the presence of the intermediate snail host, *O. hupensis*, in the region. Consequently, these cases remained unconfirmed due to the lack of robust molecular evidence. In this study, we retrieved FFPE rectal biopsy samples from four patients and successfully constructed NGS libraries from two. Through a dual-alignment strategy, we identified multiple sequences with 100% identity to *S. japonicum*, providing definitive molecular confirmation of schistosomiasis japonica in Mangshi City for the first time.

Histopathological examination remains a cornerstone in the diagnosis of parasitic and other infectious diseases. The identification of parasites or their eggs in tissue sections—typically stained with HE—is often considered diagnostic. However, distinguishing between parasite species can be challenging, particularly when morphological similarities exist or clinical manifestations are atypical. In the present study, suspected schistosomiasis cases exhibited egg-like structures in rectal biopsies, with measured dimensions ranging from 60.803 to 63.927 µm in length and 36.918–42.476 µm in width (Table 1), slightly smaller than the sizes reported for *S. japonicum* eggs [11]. This size discrepancy is likely attributable to tissue fixation and staining, which can induce shrinkage and morphological distortion. Comparative analysis with four confirmed schistosomiasis cases from endemic regions in Yunnan revealed similar egg morphologies and sizes (Supplementary Fig. 2, Additional File 7), supporting the hypothesis that histological processing may influence egg dimensions without negating diagnostic validity.

FFPE tissues are commonly archived in clinical settings and represent a valuable resource for retrospective molecular investigations. Although widely employed in oncology for mutation analysis and transcriptomics [12–14], their use in infectious disease diagnostics, particularly parasitic infections, remains limited. While high-throughput sequencing has recently enabled pathogen detection in FFPE samples, applications have primarily focused on bacterial agents [15]. Notably, Magalhães et al. [16]. detected *Fasciola gigantica* in FFPE tissues of infected *Lymnaea viatrix* via multiplex PCR, and Moradi et al. [17]. Amplified mitochondrial genes from FFPE hydatid cysts preserved for up to 2 years. However, FFPE-stored DNA degrades progressively over time, presenting significant challenges for library preparation and sequencing. In our study, the FFPE samples had been stored for 6–16 years, resulting in severe DNA fragmentation and low yields (Fig. 2C), which rendered standard sequencing approaches infeasible. To address this, we employed a micro-library construction strategy optimized for degraded DNA, enabling successful high-throughput sequencing. This approach demonstrates the feasibility of using long-preserved FFPE samples to molecularly confirm parasitic infections even after extended storage periods. Unlike conventional methods targeting a few specific genes, our genome-wide NGS strategy allows comprehensive pathogen detection, offering greater resolution for mixed infections and diagnostically complex cases.

In addition to storage duration, the DNA extraction method significantly influences the efficiency of nucleic acid recovery from FFPE tissues. Formaldehyde, the main component of formalin, induces DNA fragmentation and cross-linking with proteins, forming complexes that resist proteolytic digestion and hinder extraction. While several extraction protocols exist [18, 19], optimizing these methods for specific sample types is critical. In our initial attempts using the MagPure Soil DNA KF kit, we were unable to recover DNA. Switching to the CTAB method yielded successful extraction, underscoring the importance of selecting appropriate extraction techniques for degraded samples.

Despite these advancements, the study has several limitations. First, only two out of over 20 suspected cases were sequenced, and thus we cannot exclude the possibility that other cases may have involved different pathogens, despite similar clinical and histological features. Second, although DNA extraction was conducted in a schistosome-free laboratory, the absence of negative FFPE controls limits our ability to rule out contamination. Future studies should incorporate such controls to ensure data integrity. Third, although we confirmed *S. japonicum* infection in these patients, their atypical clinical manifestations—such as the absence of classical symptoms—may reflect low parasite burdens or strain-specific factors reducing pathogenicity. The absence of local *O. hupensis* snails in previous field surveys also remains unresolved. It is possible that the snail was never present in this area, or that ecological changes in recent years have led to its disappearance. Notably, some patients had a travel history to Myanmar, particularly Shan State, where both *S. japonicum* and *S. mekongi* have been reported [20–22]. This raises the possibility of imported infections and underscores the importance of comprehensive epidemiological investigations to clarify infection sources and evaluate potential transmission risks.

## Conclusion

This study highlights the diagnostic and epidemiological value of applying next-generation sequencing to long-preserved FFPE samples for resolving previously unconfirmed schistosomiasis cases in non-endemic regions. In Mangshi City, where no local *O. hupensis* snails were found, we identified *S. japonicum* DNA in biopsy samples stored for over a decade, confirming infections that had remained unverified. By overcoming severe DNA degradation using a micro-library construction strategy, our genome-wide approach enabled precise pathogen detection beyond the limits of conventional histology or PCR. It illustrates how retrospective molecular investigation of archived specimens can reveal hidden patterns of disease transmission, especially in the context of ecological change and human mobility. In doing so, we provide a new framework for enhancing parasitic disease surveillance and demonstrate how next-generation sequencing can support public health responses to neglected tropical diseases in both endemic and non-endemic settings.

## Supplementary Information

Supplementary material 1. Supplementary Figure 1. Distribution of read length.

Supplementary material 2. Supplementary Figure 2. Morphology of the eggs in rectal stained tissue sections from four confirmed schistosomiasis cases.

Supplementary material 3. Additional file 1. Geographical distribution of 21 suspected cases in Mangshi City, Yunnan Province, China.

Supplementary material 4. Additional file 2. Assembled sequences of sample 68938.

Supplementary material 5. Additional file 3. Assembled sequences of sample 9728319.

Supplementary material 6. Additional file 4. Details for the assembled sequences of sample 68938.

Supplementary material 7. Additional file 5. Details for the assembled sequences of sample 9728319.

Supplementary material 8. Additional file 6. Mapping of sequencing reads to human and *Schistosoma japonicum* Genomes.

Supplementary material 9. Additional file 7. Egg size statistics of confirmed schistosomiasis cases in rectal tissue sections from Yunnan Province, China.

## Data Availability

The sequencing data for the two Paraffin-Embedded Samples are available in the NCBI SRA under accession number PRJNA1261663 (https://submit.ncbi.nlm.nih.gov/subs/sra/SUB15314123/overview).

## Abbreviations

CDC: Center for disease control and prevention
FFPE: Formalin-fixed paraffin-embedded
NGS: Next-generation sequencing
CTAB: Cetyltrimethylammonium bromide
HE: Hematoxylin and eosin
