# Supplementary figures and images for: Molecular diagnosis of suspected intestinal schistosomiasis in a non-endemic area of Yunnan Province, China
Source: Infect Dis Poverty. 2025 Oct 9;14:99. doi: 10.1186/s40249-025-01372-y (PMC12512248; doi:10.1186/s40249-025-01372-y)

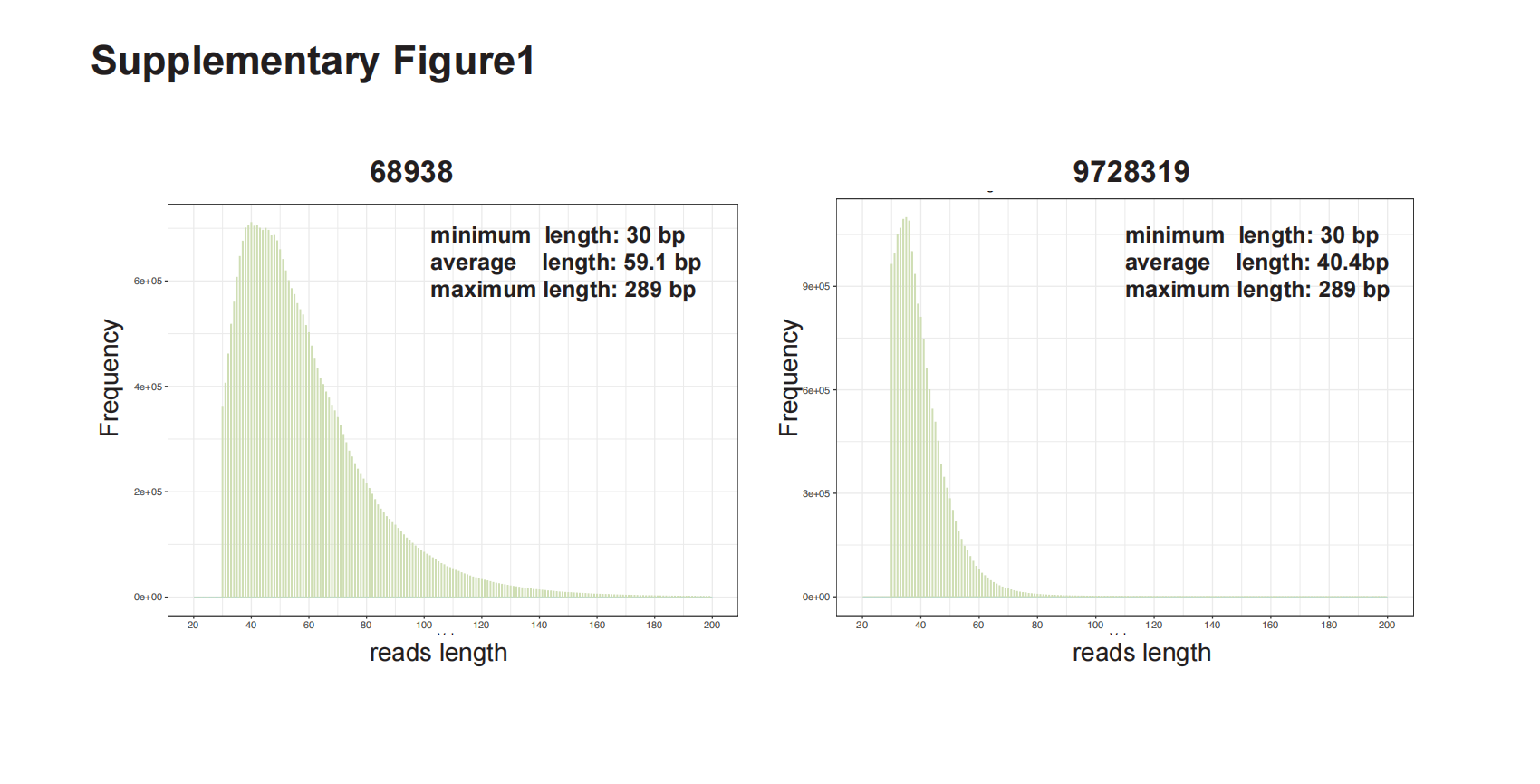

Supplement: Supplementary file 1 — Supplementary material 1. Supplementary Figure 1. Distribution of read length. [file 40249_2025_1372_MOESM1_ESM.png]

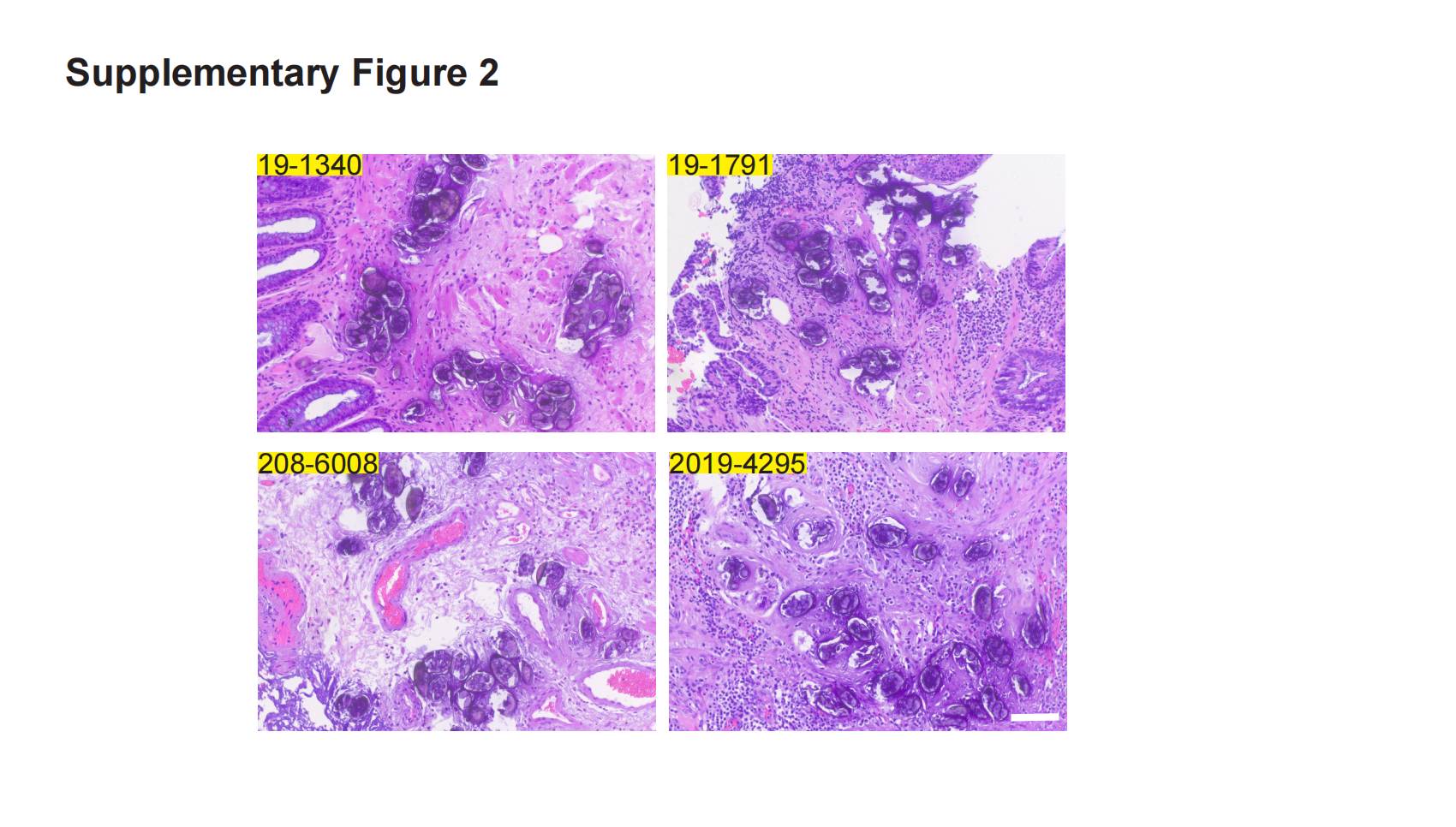

Supplement: Supplementary file 2 — Supplementary material 2. Supplementary Figure 2. Morphology of the eggs in rectal stained tissue sections from four confirmed schistosomiasis cases. [file 40249_2025_1372_MOESM2_ESM.png]
